# State of the Art in LP-WAN Solutions for Industrial IoT Services

**DOI:** 10.3390/s16050708

**Published:** 2016-05-17

**Authors:** Ramon Sanchez-Iborra, Maria-Dolores Cano

**Affiliations:** Departamento de Tecnologías de la Información y las Comunicaciones, Universidad Politécnica de Cartagena, Cartagena 30202, Spain; mdolores.cano@upct.es

**Keywords:** Low-Power Wide Area Networks (LP-WAN), Machine-to-Machine (M2M) communications, Industrial Internet of Things (IIoT), Internet of Things (IoT), wireless sensor networks

## Abstract

The emergence of low-cost connected devices is enabling a new wave of sensorization services. These services can be highly leveraged in industrial applications. However, the technologies employed so far for managing this kind of system do not fully cover the strict requirements of industrial networks, especially those regarding energy efficiency. In this article a novel paradigm, called Low-Power Wide Area Networking (LP-WAN), is explored. By means of a cellular-type architecture, LP-WAN–based solutions aim at fulfilling the reliability and efficiency challenges posed by long-term industrial networks. Thus, the most prominent LP-WAN solutions are reviewed, identifying and discussing the pros and cons of each of them. The focus is also on examining the current deployment state of these platforms in Spain. Although LP-WAN systems are at early stages of development, they represent a promising alternative for boosting future industrial IIoT (Industrial Internet of Things) networks and services.

## 1. Introduction

Machine-to-Machine (M2M) networks and Industrial Internet of Things (IIoT) services are two key enabling approaches for future industrial networking [1]. As reflected from the forecast investments predicted in the IIoT field [2], the advent of low-cost, always-connected devices opens new and exciting opportunities involving many stakeholders from a wide range of sectors. Deploying well-structured and easily-accessible M2M networks will facilitate having a precise control over the production or company’s installations, which could be translated into a smart strategy for saving logistic costs [3]. As an example, new services such as real-time event processing or 24/7 access to tracking information will be introduced into the supply chain. Having a thorough monitoring system deployed all along the manufacturing and supply chain allows enriching the complete value chain with precious information, minimizing losses against unexpected events, and hence improving both business processes and the information exchange among stakeholders (Business-to-Business (B2B) networks) [4]. In this case, smart metering (water, oil, *etc.*), goods and facilities monitoring, or smart farming are good examples of areas of activity for M2M/B2B networks.

**Figure 1:**
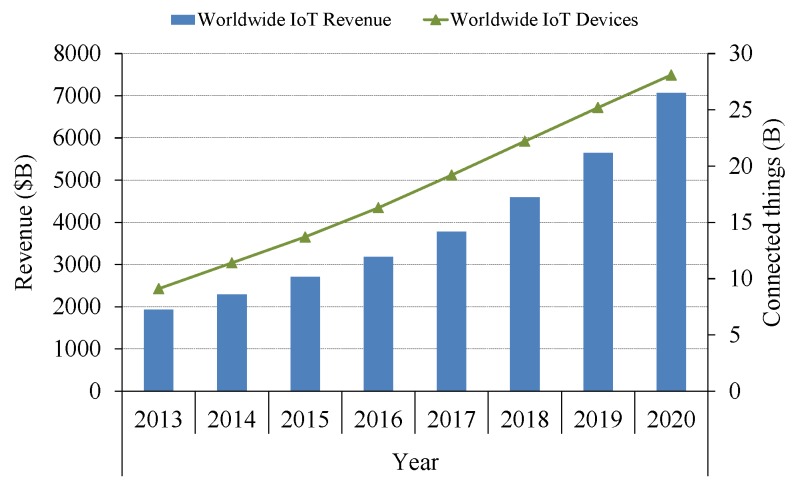
Worldwide IoT connected devices and revenues forecast. Data extracted from [5].

M2M networks can be seen as a revamp of the widely-deployed Wireless Sensor Networks (WSN); we could also think that most of the aforementioned applications are already covered by this well-studied approach. It is true that we have survived so far with the existing WSN *classic* solutions such as ZigBee, Bluetooth, or even WiFi (short-range technologies), but the main point of industrial M2M networks is the huge increase in the number of devices composing them and the notable widening of the covered areas. Global device connections are estimated to be about 28 billion by 2020 (Figure 1) [5]. This enormous growth requires (i) minimized cost per unit; (ii) optimized edge-nodes’ energy consumption; (iii) high network scalability; and (iv) wide network coverage. As discussed in the next sections, one or many of these points are the main weaknesses of traditional WSN technologies. In addition, as mentioned previously, lots of industrial applications need to operate over vast regions that are unaffordable for those *classic* WSN solutions. The need of rich coverage has been solved by means of existing cellular technologies (usually with low bandwidth), e.g., GSM (Global System for Mobile communications), GPRS (General Packet Radio Service), *etc.*, or satellite connectivity (long-range technologies), but the increased costs and the high level of power demanded by these systems make them unsuitable for long-term M2M networks composed by a massive number of devices.

**Figure 2:**
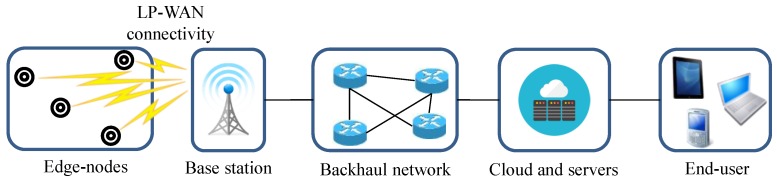
LP-WAN network architecture.

A new paradigm called Low-Power Wide Area Networking (LP-WAN) has arisen recently, aimed at filling the existing gap for deploying overcrowded M2M networks [6]. The main foundation of these systems is the deployment of highly scalable systems, usually in an operated fashion, employing low-cost edge-devices with low battery consumption. Figure 2 presents the typical architecture of a LP-WAN system. Observe that, essentially, the network architecture is similar to that of cellular networks, where one or a series of base stations provides direct connectivity from edge-devices to the backhaul network and, then, to the cloud, where the data is stored and prepared to be accessed. Regarding the edge-network architecture, it is notably different from that employed by traditional WSN. Basically, instead of composing a local network and using a gateway for sending outside the collected data, end-nodes directly connect to the base station. This configuration allows simplifying the network management complexity and also reduces energy consumption given that routing tasks are avoided.

Different LP-WAN platforms have been proposed, each of them with their own particularities and individual features that make them more suitable for different types of IIoT services. This issue will be addressed in the next sections as follows. Section 2 identifies the limitations that the *classic* IIoT solutions present. A detailed overview of the LP-WAN paradigm, covering the key characteristics of the most prominent LP-WAN platforms, is developed in Section 3. Section 4 focuses on the deployment state of LP-WAN technology in Spain. Section 5 presents a thorough discussion about the reviewed LP-WAN proposals, exploring the answers given to the challenges previously identified. Finally, the paper ends outlining the main conclusions.

## 2. Limitations on Existing IIoT Solutions

Current enabling technologies for IIoT services can be divided into short-range and long-range approaches. The main impediments found to implement sustainable cost-effective IIoT solutions are related to: (i) network management costs; (ii) scalability and network organization; (iii) edge-nodes’ dimensioning and power efficiency; and (iv) coverage. In the following, these points are identified and reviewed for different short-range and long-range technologies that have been employed so far for supporting IIoT applications. Please note that although the list of solutions provided in this section does not intend to be exhaustive, it permits us to identify the principal challenges in deploying these types of M2M networks.

### 2.1. Short-Range Connectivity

Systems with short-range connectivity were the first ones employed to manage WSN. Depending on the adopted wireless technology, which strongly determines the Physical (PHY) and Medium Access Control (MAC) layers, the network presents more suitable characteristics for supporting one application or another.

Regarding the network management costs, one typical characteristic for this kind of solution is the private ownership of a great part of the network. This fact should not be ignored because it causes an increase in both the expense and complexity of the operations. On the one hand, the owner is in charge of the complete deployment process, from the edge-device placement to the backhaul network management, in order to make data accessible from outside (including security issues). Besides, failures happening in the private part of the system should be handled by the owner company, which might not be always be able to cope with these tasks and would have to assume extra expenses by outsourcing this service. On the contrary, by employing public networks, there is a clear change in the business model and, hence, the deployment costs are shared: the subscriber assumes the edge-device costs, whereas the network operator bears the backhaul network deployment and maintenance expenses. During the operation stage, the subscriber pays a fee to the network operator for the system maintenance service, some kind of technical support, and, usually, for having a friendly back-end for data accessing. In the case that a traditional WSN adopted the public-architecture strategy, the direct communication between both extremes would not be feasible due to the limited transmission range of the edge-nodes [7]. Thus, additional equipment, *i.e.*, gateways, or sophisticated data-collection strategies, would be needed to connect the edge-nodes to the central base station.

In addition to network management, as a large-scale issue, there are other problems regarding the edge-nodes’ functionality when they are managed by the existing short-range solutions. For example, the most employed technologies for operating WSNs, *i.e.*, the IEEE 802.15.4-based protocols ZigBee and 6loWPAN, present highly interesting features in terms of energy efficiency and the low cost of the edge-devices. However, the growth of this type of network is limited because the management complexity and interference issues could suffer a noticeable increase with the increment of the network size [7,8]. Although several routing algorithms based on different paradigms such as multi-hop routing, opportunistic networks, or delay-tolerant networks have been proposed, an important number of concentrators (or information collectors) might be still needed in relatively large networks, which could also increase the overall network power consumption [9].

As well as the possible effect in terms of higher network consumption in more dense scenarios with ZigBee or 6loWPAN protocols mentioned before, the use of other technologies such as WiFi and Bluetooth (not oriented to WSN in its inception, but widely used for this purpose) could have a negative impact on energy efficiency. The main issue presented by these solutions is that they were designed to support highly-bandwidth-demanding applications and, hence, transmission/reception tasks waste a lot of energy. Additionally, the management of a network composed by a significant number of nodes is also tricky as these networks are often based on the Internet Protocol (IP), so different topology-organization methods, e.g., clustering, are needed [10,11].

Another important issue, common to all short-range technologies mentioned so far (IEEE 802.15.4-based protocols ZigBee and 6loWPAN, WiFi, and Bluetooth), is the need for a connection to the Internet in order to upload all collected data to the cloud. While in urban or suburban areas this should not be a problem, in remote locations it could be difficult or, at least, expensive because these areas usually lack a preexisting infrastructure that could provide Internet access [12]. Additionally, special equipment such as bridges is needed for different reasons. Firstly, these nodes are employed as intermediate points between the backhaul network and the edge-nodes due to the limited coverage range of the latter. Besides, all the collected data need to be gathered and formatted before sending it to the storage servers. When talking in terms of Big Data, accomplishing an accurate dimensioning of the bandwidth and the temporal storage needs of these devices is not a trivial task. For all these reasons, other approaches based on long-range technologies have also been employed for deploying IIoT services.

### 2.2. Long-Range Connectivity

The first idea that comes to mind in order to solve the issues described above is cellular networks: they are based on public infrastructure, they are widely deployed and cover large areas, and they are operated employing well-known standards such as GSM, GPRS, or 3G/4G. Following this strategy, the edge-sensors collect the data of interest and, afterwards, send it to the cloud via a cellular data link, e.g., GPRS, 3G, *etc.* However, the main problem with these systems is that they were designed to fulfill different requirements than those of IIoT services. While in cellular networks the trend has been increasing the available bandwidth, aiming to accomplish the increasing demand of multimedia traffic by human users [13], in IIoT services the strategy should be optimizing bandwidth usage and decreasing energy consumption and costs [14]. Current cellular base stations are capable of hosting a small number of connected users (in comparison with the needs of sensorization services), with a relatively high bandwidth assured for each of them. In turn, what a machine-only network demands is a solution for supporting a huge number of low-throughput connected devices that send short messages only once in a while. Therefore, the current cellular solutions are clearly inefficient in terms of scalability and energy consumption. Regarding the former, one possible strategy for organizing and providing connectivity to independent systems is using femtocells [15] or picocells [16]. However, this solution notably increases the system cost as new equipment and connection infrastructure are required. Focusing on energy efficiency, cellular networks need a quasi-constant communication between edge-nodes and the base station for management tasks (protocol overhead), which is completely devastating for battery lifetime. Moreover, existing cellular networks work on scarce and expensive (licensed) frequency bands.

Another solution with even more drawbacks is satellite communications. Although they provide a good coverage worldwide, the energy consumed in each transmission is too much for IIoT applications. In addition, the high latency of these transmissions could be inadmissible for certain applications with strict temporal constraints. Finally, with respect to network costs, subscribing a satellite connection plan is still excessively expensive. Although cheaper, current cellular network operators have not substantially reduced their subscription fees. For all these reasons, Low-Power Wide Area Networks appear as an alternative long-range solution to give response to the IIoT services’ demands.

## 3. LP-WAN Solutions for IIoT Services

**Figure 3:**
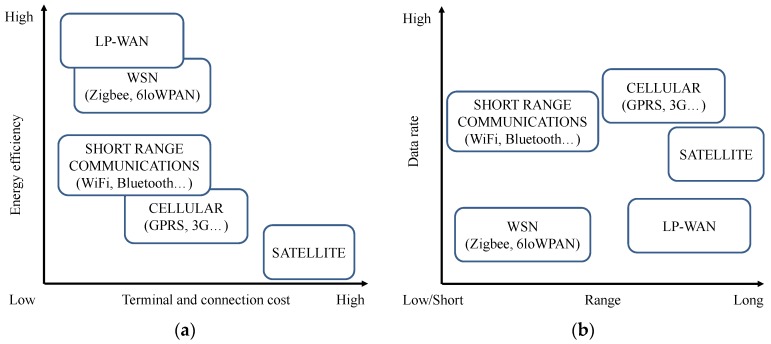
Principal characteristics of IIoT-enabling technologies. (**a**) Data rate and coverage range; (**b**) Energy efficiency and terminal and connection cost.

Recently, a number of different platforms following the LP-WAN paradigm have arisen. These proposals aim at gathering both the long transmission range provided by cellular technologies and the low energy consumption of WSNs (Figure 3). Many LP-WAN proposals are at an early development stage and others have already begun their architecture deployment. LoRaWAN, Sigfox, and Ingenu are currently the LP-WAN platforms with the greatest momentum and they have been reviewed in recent works [17,18]. However, there are many other proprietary and standard platforms with interesting proposals that we also consider in the following sections. Although each of these LP-WAN solutions has its own particularities and protocols (many of them proprietary), there are some common foundations which all of them rely on.

As shown in Figure 2, LP-WANs make use of a star topology, where all edge-nodes are directly connected to the base station; hence, the LP-WAN modem is directly installed in edge-devices. In some cases, concentrators/gateways can be used to connect a cluster of nodes to the base station (star-of-stars topology). The base station and the backhaul network are usually public and operated by the service provider. As discussed above, this fact liberates subscribers from deployment, maintenance tasks, and operational costs related to this part of the system. Regarding the edge-network connectivity with the base station, most of the proposed platforms employ ISM (Industrial, Scientific, and Medical) frequency bands; concretely, the most employed frequencies are those within the sub-GHz bands, namely 868 MHz in Europe, 915 MHz in the US, and 920 MHz in Japan. In comparison with the 2.4 GHz band, transmitting in a lower-frequency band leads to a deeper wave penetration and range, which are highly valued characteristics in order to provide indoor connectivity. Furthermore, electronic circuits are more efficient at lower frequencies.

Another common characteristic in these systems is the asymmetric connectivity provided to edge-nodes. Aimed at reducing energy consumption, most of the solutions focus on the uplink connection; thus, the downlink is severely limited, hence reducing the necessary “listening” time needed for receiving data. It is clear that most data flow from the edge-network to the core, but in the case of having not only sensors but also actuators, an effective downlink would be also highly appreciated. It would be useful for updating the edge-nodes’ software, too. To deal with these issues, different strategies have been adopted to provide a base station-to-edge-nodes downlink, as discussed later.

In summary, the main advantages that all LP-WAN platforms claim to own are: (i) high scalability and range, necessary for super-crowded networks deployed in vast areas; (ii) roaming, useful for goods-delivery tracking; (iii) real-time event alerts, which are set up by the customer and automatically triggered from the LP-WAN operator’s management system; and; (iv) low edge-node energy consumption and cost. In the following, a brief review about the most prominent LP-WAN platforms arisen so far is provided.

### 3.1. LoRaWAN

This platform is promoted by the LoRa Alliance [19], composed by IBM, Semtech, and Actility, among others. It proposes a star-of-stars topology with dedicated gateways serving as transparent bridges between edge-nodes and the central network, where the data is stored and made available to the subscriber. The edge-nodes connect to the access points via one-hop links by using the LoRa (Long Range) modulation. This is Semtech’s proprietary Chirp Spread Spectrum (CSS) radio scheme that employs a wide channel of up to 250/500 kHz (Europe/North America) and provides adaptive data rate capabilities by means of a variable processing gain. Please note that this concept represents the ratio between the chip rate and the baseband information rate, and is usually known as the Spreading Factor (SF). LoRaWAN presents a SF from 7 to 12. Using this last characteristic, edge-nodes can tune the transmission power and bitrate to the real network conditions, allowing a reduction in energy consumption. Moreover, LoRaWAN defines three types of edge-devices depending on their downloading needs: Class A devices have a scheduled downloading window just after each uplink connection (Receiver-Initiated Transmission strategy, low power consumption), Class B devices have additional scheduled downlink windows (Coordinated Sampled Listening strategy, medium power consumption), and Class C devices can receive messages almost at any time (Continuous Listening strategy, large power consumption). In its specification sheets, LoRaWAN claims a Class A edge-node’s battery lifetime is over five years.

Originally, LoRaWAN was designed to work in ISM bands but it can be also adapted for supporting the licensed spectrum. Under these conditions, LoRaWAN claims to demodulate signals 19.5 dB below the noise floor, hence achieving greater ranges than those provided by cellular base stations. In both communication directions, the adaptive data-rate ranges from 0.25 kbps (0.98 kbps in North America due to FCC (Federal Communications Commission) limitations) up to 50 kbps, with a maximum payload length of 256 bytes. Finally, security issues have been thoroughly considered, so that end-to-end AES (Advanced Encryption Standard) encryption security, including the use of a unique network, application, and device keys for encrypting data at different OSI (Open Systems Interconnection) levels, is provided.

### 3.2. Sigfox

This is the platform in the most advanced deployment state in Europe. By means of agreements with local cellular network operators, Sigfox [20] claims to have covered most of the territory of France, Russia, and Spain, among others. Technically speaking, this solution is quite different from the LoRaWAN approach. Instead of using bidirectional spread spectrum channels, Sigfox employs proprietary ultra-narrow band modulation (Differential Binary Phase Shift Keying, DBPSK) with a heavily limited uplink connection. Using this modulation, a maximum data rate of 100 bps can be achieved by transmitting messages with a maximum payload length of 12 bytes. Meanwhile, using this low bitrate permits large ranges of 10 km and beyond with very low transmission power, which allows saving energy at edge-nodes. Sigfox’s technical sheets claim a typical stand-by time of 20 years with a 2.5 Ah battery.

Sigfox’s star topology is similar to a cellular architecture, with a wide deployment of base stations aimed at covering entire countries by employing ISM bands. This base station structure permits edge-nodes to upload the gathered data directly to Sigfox servers, which makes it accessible to subscribers through a web-based API (Application Programming Interface). The use of ISM bands together with Sigfox’s medium access strategy, namely without collision-avoidance techniques, leads to a stringent bandwidth-occupancy limitation suffered by edge-nodes. For example, a duty cycle of 1% is established in the Europe regulations; hence, a maximum of 140 messages per edge-node per day are allowed. In the case of the USA regulations, Sigfox’s limited data rate of 100 bps shows that transmitting single messages usually takes 2–3 s, which is outside the FCC’s maximum message transmission time in ISM bands of 0.4 s. Although originally designed as a unidirectional system, Sigfox has lately included a limited downlink window (four messages of eight bytes per edge-node per day) similar to the strategy adopted by LoRaWAN’s Class A devices (please see previous sub-section).

Regarding security issues, Sigfox implements frequency-hopping and anti-replay mechanisms in their servers, but no encryption techniques are used between end-nodes and base stations. Additionally, the payload format is undefined. Therefore, Sigfox’s security strategy relies on the fact that an intercepted message cannot be interpreted unless the attacker is able to understand the particular subscriber’s system.

### 3.3. Weightless

Weightless is the alliance name for a set of three LP-WAN open standards: Weightless-W, Weightless-N, and Weightless-P [21]. The three Weightless flavors work in sub-GHz bands, but each of them has its own particularities.

The original Weightless-W standard makes use of the TV whitespace spectrum and provides a wide range of modulation schemes, spreading factors, and packet sizes. Considering all these features, and depending on the link budget, Weightless-W claims to achieve two-way data rates from 1 kbps to 10 Mbps with very low overhead. Due to the extensive feature set provided by Weightless-W, the edge-node’s battery lifetime is limited to three years and the terminal cost is higher than that of its competitors. The communication between the edge-nodes and the base station can be established along 5 km, depending on the environmental conditions.

In turn, Weightless-N uses a class of low-cost technology, very similar to that employed by Sigfox. Thereby, ultra-narrow band (DBPSK) modulation is adopted in order to provide unidirectional-only connectivity of up to 100 bps, exploiting ISM bands. This scheme is based on nWave’s technology [22], which was donated as a template for the Weightless-N standard. Because of the simplicity of this solution, Weightless-N allows a battery duration of up to 10 years, very low cost terminals, and a long connection range similar to that reached by Weightless-W.

Finally, the newest Weightless-P open standard is derived from the M^2^Communication’s Platanus protocol [23]. This version gathers together the most proper characteristics of the previous standards, and it claims to be specifically focused on the industrial sector. Using a narrow-band modulation scheme (Gaussian Minimum Shift Keying, GMSK, and Offset Quadrature Phase Shift Keying, OQPSK) operating in 12.5 kHz channels, Weightless-P implements bi-directional communication with an adaptive data rate from 200 bps to 100 kbps. It supports both ISM and licensed spectrum operation. Aimed at providing the reliability demanded by some industrial applications, Weightless-P includes, by default, valued characteristics such as acknowledged transmissions, auto-retransmission, frequency and time synchronization, and channel coding, among others. Compared with the other Weightless standards, Weightless-P provides a more limited range of 2 km and its advanced features in comparison with Weightless-N permit a shorter battery lifetime of three years.

Regarding security, the three Weightless versions provide end-to-end network authentication and 128 bit AES encryption.

### 3.4. Other Alternatives

Besides the three solutions mentioned so far, there are other alternatives that, up to the date of preparing this article, either are in a less advanced deployment state or their technical insights are not yet available. For example, Ingenu (formerly known as On-Ramp) is a LP-WAN platform currently beginning its deployment in the USA. It is based on its proprietary RPMA (Random Phase Multiple Access) technology, which has the particularity of working in the 2.4 GHz band. In addition, it permits both star and tree topologies by using different network hardware. Although Ingenu has raised high expectations regarding the range, edge-device’s battery lifetime, and available bandwidth [24], these promising figures should be confirmed in real deployments as they have been extracted so far only from simulation studies.

Mostly focused in the Smart Cities market, Telensa [25] has also developed its own bi-directional ultra-narrow-band technology. Telensa’s PLANet (Public Lighting Active Network) and PARKet are focused on street lighting control and smart parking enhancement, respectively. Both of them are defined as end-to-end systems, from edge-nodes (telecells) to the end-user interface, including base stations. By using their proprietary technology, Telensa claims to reach 2–3 km (urban) and 5–8 km (rural) real ranges. They have already deployed their solutions in different big cities worldwide.

In turn, Dash7 is an open standard promoted by the Dash7 Alliance [26], which has its origin in the ISO/IEC 18000-7. Unlike the afore-reviewed solutions, Dash7 proposes a two-hops tree topology composed by hierarchized devices, namely endpoints, sub-controllers, and gateways. Notice that this topology is similar to the traditional WSN architecture instead of the long-range systems described in this article. The main advantages provided by the Dash7 protocol are the extended range in comparison with other pure-WSN solutions due to the use of sub-GHz bands (433 MHz and 868/915 MHz), the possibility of direct device-to-device communication, which is not currently available in any of the LP-WAN platforms described above, and its compatibility with Near Field Communication (NFC) radio devices. However, this proposal has not been widely adopted yet, and only some pilot projects have been carried out so far [27].

Finally, it is worth mentioning other solutions such as those proposed by Helium [28], M2M Spectrum Networks [29] (recently joined the LoRa Alliance), or Amber Wireless [30] which, although less expanded, could bring more competence to this growing market in the future.

### 3.5. Standardization Bodies’ Efforts

Besides the platforms reviewed above, there are different solutions proposed by well-recognized standardization bodies that are currently under study. For example, the IEEE has proposed the P802.11ah [31] and 802.15.4k [32] standards. The former presents a series of modifications at the 802.11 PHY and MAC layers aimed at adapting them to sub-GHz bands (excluding TV white space). Using the well-studied Orthogonal Frequency Division Multiplexing (OFDM), it is intended to reach a minimum data rate of 100 kbps and a transmission range up to 1 km [33]. In this standard, the co-existence with other technologies, such as all those based on the IEEE 802.15.4 PHY-layer specifications, is being considered. In turn, the IEEE 802.15.4k standard presents MAC and PHY layer specifications to facilitate Low Energy Critical Infrastructure Monitoring (LECIM) applications. This standard defines two PHY modes: Direct-Sequence Spread Spectrum (DSSS) and Frequency Shift Keying (FSK). The former permits links of up to 20 km in line of sight (5 km in non–line of sight) with data rates of up to 125 kbps. The proposed architecture is a point-to-multipoint network by means of a star topology composed by two types of nodes, namely a PAN (Personal Area Network) coordinator and the edge-devices. The communication between the collector and the sensors is asymmetric, aimed at limiting the “listening” time of the battery-powered sensors. This standard permits employing both sub-GHz and 2.4 GHz bands using Binary Phase Shift Keying (BPSK) and OQPSK modulations.

In turn, the 3GPP group (3rd Generation Partnership Project) is working on the development of the LTE-MTC (Long Term Evolution-Machine-Type Communications) standard [34]. In the LTE Release 12, the Cat 0 speed of 1 Mbps was defined, but in order to reduce the chipset’s complexity and power consumption, there is a plan to define an even lower speed of about 200 kbps (referred to as Cat M) in the next release, Release 13. Although the standard is still being developed, it has been decided to make use of 1.4 MHz channels within the cellular band (450 MHz) in order to provide bi-directional connectivity between edge-nodes and the base station. Finally, aimed at presenting a comprehensive comparison among all the reviewed LP-WAN platforms, Table 1 shows their most relevant characteristics. Please note that the presented values have been extracted from the platform’s specification sheets and some of them could be provisional figures due to the ongoing evolution of the different solutions.

## 4. Current Deployment State of LP-WAN Solutions in Spain

As in the rest of the world, the rollout of LP-WAN platforms in Spain is in its beginning stages. Currently, there is one solution with a clear advantage over the rest: Sigfox. After reaching an agreement with the network operator Cellnex Telecom [35,36], Sigfox has reached a count of more than 1300 base stations covering the Spanish territory. Thus, Sigfox employs the already-deployed Cellnex (previously known as Abertis Telecom) infrastructure. This strategy of partnering with a big network operator has been also adopted by Sigfox in other countries such as France (TDF [37]) and the Netherlands (Aerea [38]). Regarding the Spanish case, Sigfox has focused on security services (e.g., to connect alarm systems to the cloud) and is beginning its expansion to other niche markets (e.g., in smart farming and precision agriculture).

Although far from the Sigfox network’s deployment state, other platforms have begun their landing in Spain, too. For example, a LoRaWAN pilot network is planned to be deployed in the city of Malaga by the Swiss company iSPHER [39,40]. Therefore, by rolling out their SPHER NET, an operational end-to-end LoRa IoT network solution, the full city territory will be covered. Up to the date of writing this article, this project is still at an early stage of development.

Regarding the standard solutions, the deployment of the LTE-MTC technology will permit current cellular carriers to take advantage of their deployed infrastructure. LTE-MTC will be compatible with the normal construct of LTE networks, so the network operators only will have to update their systems’ software. In Spain, several cellular carriers have already deployed their own infrastructure; thus, more competitors will arise with the advent of this promising standard.

Aimed at providing a specific scenario of applicability for LP-WAN solutions, in the following the case of irrigation water smart metering is discussed; this is a greatly valued good in the southern regions of Spain [41,42]. Due to the shortage of water and its expensive price, both water companies and farmer associations are highly interested on having a thorough control of water consumption [43]. The main obstacle found until now is the remote location of the fields, which in many cases lack of any kind of connectivity or even electricity. Therefore, having a centralized control of water consumption is greatly challenging in this scenario. Due to the great distances among fields, it is not feasible to deploy an interconnected WSN with the aim of routing the collected to data to a gateway connected to the Internet. Even more, as explained in previous sections, the gateway’s Internet connection would be difficult and expensive to establish. In such remote locations, it is usual to not have GSM/GPRS coverage, so employing cellular networks is not a valid strategy either. Therefore, this is a good example of the applicability of LP-WAN solutions. Given the great coverage range of base stations, especially in free space, one of these stations can provide connectivity to several water meters, which can directly submit their readings to the base station, making them accessible almost in real time. Thus, abusive consumption, water theft, or pipe losses can be easily detected, increasing the whole system’s efficiency with an inexpensive investment [44].

## 5. Discussion (All that Glitters Is Not Gold)

We are witnessing the dawn of LP-WAN solutions for wide and overcrowded M2M networks and IIoT services. There are differentiating characteristics such as the data rate, power consumption, or cost that work against each other. Consequently, none of the existing platforms provides the best performance for all of these requirements. Thus, once the needs of the service to be deployed are specified, the LP-WAN solution that matches best will be chosen. For that reason, there is not a clear dominant platform yet among all the arisen platforms that could completely fulfill the key challenges identified in Section 2:

- Focusing on management costs, most platforms offer the same model to their customers: the subscriber assumes the expenses of deploying the edge-network and pays a fee to the LP-WAN operator for managing and making all the collected data accessible. This is an adequate solution, as the issues and expenses related to the information management process are avoided by the subscriber.
- In terms of network organization and the edge-nodes’ dimensioning, it seems that the star topology allows an easy and straight connection from each end-node to the base station. However, although all the cited solutions claim high system scalability with base station capacities of thousands of simultaneously connected nodes, other topologies such as star-of-stars or tree architectures could improve this scalability at the expense of employing special nodes (concentrators) and increasing the edge-network complexity.
- Regarding power efficiency, every reviewed platform ensures edge-node lifetimes of some years. Of course, these figures depend on the number of messages transmitted per day, the transmission bitrate, and other factors such as the edge-node’s downlink strategy.
- Concerning the area covered by the system, the explored solutions claim connectivity ranges of at least 1 km from the base station. Those platforms operating at the sub-GHz band take advantage of greater transmission distances and wave penetration in comparison with those systems employing the 2.4 GHz band. In addition, solutions adopting a hierarchized architecture, e.g., Dash7, could also extend the network coverage at the expense of needing more hops between the edge-nodes and the backhaul network.

Furthermore, there are other points regarding the service reliability and security that seem important for the proper operation of IIoT applications and represent challenges not fully covered yet. Focusing on reliability, it is clear that outdoor or industrial environment conditions are not the most favorable for sensor (edge-device) deployment. They are sometimes installed in extreme temperature and moisture conditions, near potential noise (acoustic and electromagnetic) sources, or under other hostile scenarios. Considering that M2M networks are self-regulated and that one unheard or non-transmitted message could provoke loss of revenues, the reliability of these systems should be heavily ensured. In addition, most of the cited platforms avoid using the 2.4 GHz band because of its “current saturation” [45]. However, in the near future the forecasted billions of connected things will be transmitting in the sub-GHz band; hence, the impact of the interferences among all the co-existing technologies will not be negligible either. As another relevant point, the sending and processing time for each transmission should not be ignored in applications with severe timing constraints or in the case of messaging between sensors and actuators. Besides, an effective downlink should be ready to transmit the proper message back to the edge-network if necessary. In architectures where direct device-to-device communication is allowed, e.g., Dash7, this issue could be easily solved, but in the more common star topology, messages should be firstly processed by the LP-WAN operator’s systems.

Regarding security, for mission-critical or high-security applications, the use of private data storage or servers would be more convenient than using third-party (e.g., LoRaWAN, Sigfox, *etc.*) servers. In the last case, the data owner could lose control of the information management process; this could be risky or even unacceptable in certain applications. Additionally, as the ISM bands are freely accessible, they are vulnerable to a broad range of security threats; therefore, including extra functionality to support the functions of confidentiality, authentication, authorization, or even accounting would be very welcome. Of course, all these new features would be against the edge-device’s power consumption, so a balance between the edge-nodes; functionality and energy use would be necessary.

Besides these important issues more focused on the network’s technical insights, the business model emerges as another key challenge for taking advantage against the competitors. Having the best technological solution does not always lead to success. For example, we have seen that ultra-narrow-band technology presents a series of drawbacks in comparison with other modulation schemes that offer better connectivity. However, Sigfox seems to be very attractive to potential customers due to its simplicity and its higher degree of deployment. It is on this last point where LP-WAN companies have to make the biggest economical effort and some of them have focused on different specific regions. While Sigfox seems to be more focused, for the moment, in Europe, with several countries fully covered, LoRa-WAN and Ingenu are focused on the North American market. Regarding territory coverage plans, they are commonly designed regarding the territory’s population; thus, the major urban areas are usually mostly covered but there is often a lack of connectivity in rural sites. Precisely, many big factories and farms are isolated in these emplacements, so quasi-dedicated base stations will be needed to provide services to these customers.

To sum up, we are currently in a highly dynamic scenario, with all the different platforms positioning themselves in the market. The diverse technological and business solutions offered by each of them will determine their success or failure, but there is no doubt that the LP-WAN is a rising technology that will play an important role in the forthcoming expansion of IIoT services.

## 6. Conclusions

This article discussed different enabling solutions for the imminent IIoT era. Taking advantage of these technologies will make companies ready to tackle future large-scale challenges, improving business productivity at several levels. In addition, the new networking solutions presented here are also focused on reducing power consumption in order to construct more efficient and sustainable architectures. The LP-WAN paradigm seems to be a promising response to the limitations showed by current technologies, but we are just at the very beginning of the IIoT explosion, so it will be necessary to remain vigilant to the new challenges that the upcoming M2M-based services will pose.

## Figures and Tables

**Table 1:** LP-WAN platforms summary.

	LoRaWAN	Sigfox	Weightless			Ingenu	Telensa	Dash7	IEEE 802.15.4k (DSSS)	IEEE P802.11ah	LTE-MTC
			-W	-N	-P						
Band	433/868/780/915 MHz	868/915 MHz	TV whitespace	Sub-GHz	Sub-GHz	2.4 GHz	Sub-GHz	Sub-GHz	Sub-GHz/2.4 GHz	Sub-GHz	Cellular
Max. data-rate	50 kbps	100 bps	10 Mbps	100 bps	100 kbps	19 kbps/MHz	346 Mbps	-	125 kbps	346 Mbps	200 kbps
Range (urban)	5 km	10 km	5 km	5 km	2 km	15 km	1 km	3 km	5 km	1 km	5 km
Packet-size	Max. 256 B	12 B	Min. 10 B	Max. 20 B	Min. 10 B	Max. 10 kB	Max. 65 kB.	-	Max 32 B	Max. 65 kB.	-
Downlink	Yes. Different plans	Yes (not sym.)	Yes (sym.)	No	Yes (sym.)	Yes (not sym.)	Yes (sym.)	Yes (sym.)	Yes (not sym.)	Yes (sym.)	Yes (sym.)
Topology	Star-of-stars	Star	Star	Star	Star	Star/Tree	Star/Tree	Star	Star	Star/Tree	Star
Roaming	Yes	Yes	Yes	Yes	Yes	Yes	Yes	Yes	-	Yes	Yes
Security	Fully addressed	Partially addressed	Fully addressed	Fully addressed	Fully addressed	Fully addressed	In development	-	Partially addressed	In development	In development
Protocol ownership	Partially proprietary	Proprietary	Standard	Standard	Standard	Proprietary	Standard	Proprietary	Standard	Standard	Standard

## Abbreviations

The following abbreviations are used in this manuscript:

LP-WAN	Low-Power Wide Area Networking
M2M	Machine-to-Machine
IIoT	Industrial Internet of Things
B2B	Business-to-Business
WSN	Wireless Sensor Networks
GSM	Global System for Mobile communications)
GPRS	General Packet Radio Service
MAC	Medium Access Control
ISM	Industrial, Scientific, and Medical
CSS	Chirp Spread Spectrum
SP	Spreading Factor
FCC	Federal Communications Commission
DBPSK	Differential Binary Phase Shift Keying
GMSK	Gaussian Minimum Shift Keying
OQPSK	Offset Quadrature Phase shift Keying
RPMA	Random Phase Multiple Access
LECIM	Low Energy Critical Infrastructure Monitoring
3GPP	3rd Generation Partnership Project
LTE-MTC	Long Term Evolution-Machine-Type Communications
